# Synthesis and Therapeutic Applications of Iminosugars in Cystic Fibrosis

**DOI:** 10.3390/ijms21093353

**Published:** 2020-05-09

**Authors:** Anna Esposito, Daniele D’Alonzo, Maria De Fenza, Eliana De Gregorio, Anna Tamanini, Giuseppe Lippi, Maria Cristina Dechecchi, Annalisa Guaragna

**Affiliations:** 1 Department of Chemical Sciences, University of Napoli Federico II, via Cintia, 80126 Napoli, Italy; anna.esposito5@unina.it (A.E.); daniele.dalonzo@unina.it (D.D.); maria.defenza@unina.it (M.D.F.); 2 Department of Molecular Medicine and Medical Biotechnology, University of Napoli Federico II, via S. Pansini 5, 80131 Napoli, Italy; edegrego@unina.it; 3 Laboratory of Molecular Pathology-Department of Pathology and Diagnostics, University Hospital of Verona, 37126 Verona, Italy; anna.tamanini@aovr.veneto.it; 4 Department of Neurosciences, Biomedicine and Movement, Section of Clinical Biochemistry, University of Verona, 37134 Verona, Italy; giuseppe.lippi@univr.it (G.L.); mcristina.dechecchi@gmail.com (M.C.D.)

**Keywords:** cystic fibrosis, cystic fibrosis transmembrane conductance regulator (CFTR), iminosugars, miglustat, glycomimetics, glycosidase inhibitors, CFTR correctors, anti-inflammatory agents

## Abstract

Iminosugars are sugar analogues endowed with a high pharmacological potential. The wide range of biological activities exhibited by these glycomimetics associated with their excellent drug profile make them attractive therapeutic candidates for several medical interventions. The ability of iminosugars to act as inhibitors or enhancers of carbohydrate-processing enzymes suggests their potential use as therapeutics for the treatment of cystic fibrosis (CF). Herein we review the most relevant advances in the field, paying attention to both the chemical synthesis of the iminosugars and their biological evaluations, resulting from in vitro and in vivo assays. Starting from the example of the marketed drug NBDNJ (*N*-butyl deoxynojirimycin), a variety of iminosugars have exhibited the capacity to rescue the trafficking of F508del-CFTR (deletion of F508 residue in the CF transmembrane conductance regulator), either alone or in combination with other correctors. Interesting results have also been obtained when iminosugars were considered as anti-inflammatory agents in CF lung disease. The data herein reported demonstrate that iminosugars hold considerable potential to be applied for both therapeutic purposes.

## 1. Iminosugars: Powerful Glycomimetics

**Figure 1:**
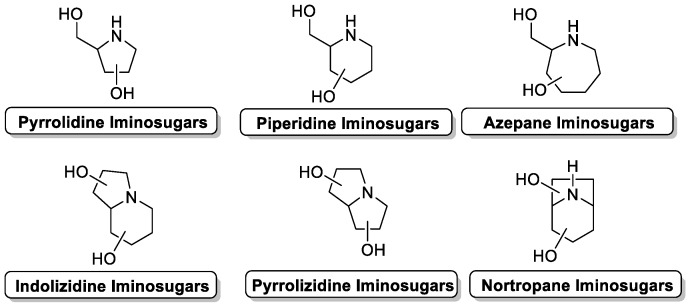
Iminosugar structural motifs of natural and synthetic origin.

Whether of natural (from plant or microbial world) or synthetic origin, iminosugars contain a wide variety of small organic molecules belonging to the class of pyrrolidines, piperidines, azepanes, nortropanes, pyrrolizidines and indolizidines (Figure 1) [1].

**Figure 2:**
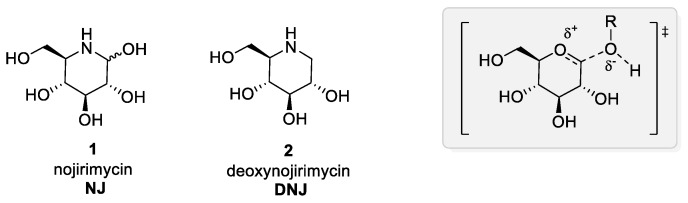
Iminosugars: carbohydrate mimetics.

After the isolation from bacteria (*Streptomyces*) of the first natural iminosugar, nojirimycin (NJ, **1**) [2], the chemical synthesis and isolation from mulberry of deoxynojirimycin (DNJ, **2**) [3], a potent α- and β-glucosidase inhibitor [4], opened the way to the advent of this new class of glycomimetics. In their simplest structure, they typically mimic the corresponding furanose or pyranose monosaccharide skeleton by replacement of endocyclic oxygen with an amino function, resembling carbohydrate substrates or saccharide hydrolysis transition states (Figure 2) [5,6,7].

Thanks to their simple and small structure strictly related to carbohydrates, iminosugars are endowed with an excellent drug profile. Indeed, they are water soluble and therefore orally administrable, they show an efficient uptake [8], taking in some cases advantage from transport mechanisms devoted to handling of carbohydrates. The ability of iminosugars to cross the blood–brain barrier can be ascribed to the same cellular mechanisms [1]. However, because most iminosugars typically lack the acetal function of carbohydrates, they are chemically and biologically stable during the main processes carried out by carbohydrate-modifying enzymes, allowing their unchanged excretion essentially in urine.

The first recognized property of iminosugars was their ability to act as glycosidase [5,7] and glycosyltransferase inhibitors [9,10], essentially acting in a competitive manner and exerting their activity mainly as antivirals (HIV-1, HSV, BVDV, HCV) [11,12] and antidiabetics [13,14,15,16]. Furthermore, they were also found to inhibit other classes of enzymes, such as metalloproteinases [17], sugar nucleotide mutase or nucleoside-processing enzymes [18,19,20].

Over the last decades, iminosugars have become the most popular class of carbohydrate-processing enzyme modulators, being able to alter the glycosylation profile of eukaryotic cells, to interfere in carbohydrate and glycoconjugate metabolism, to regulate the folding and transport of glycoproteins, and to stop the interaction mediated by host cell carbohydrates with infective agents. All these abilities allowed relevant applications of these small molecules as immune system modulators [21,22,23], anti-cancer agents [24] and as therapeutic agents for the treatment of lysosomal storage disorders [25,26,27,28,29], a group of more than 50 rare disorders caused by specific mutations in genes encoding lysosomal enzymes and transporters.

**Figure 3:**
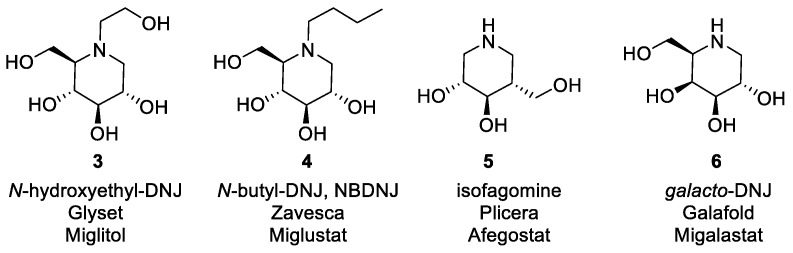
Marketed iminosugars.

Among the first-generation iminosugars, Glyset^®^ (**3**, Miglitol; Figure 3) [30,31] was the first to reach the market (Bayer), as it was approved in 1996 for the treatment of non-insulin-dependent (type II) diabetes mellitus owing to the ability to control glucose absorption from the gut through intestinal α-1,4-glucosidase inhibition, reducing the carbohydrate breakdown in the upper gastrointestinal tract. Unfortunately, closely related to its mechanism of action, the onset of side-effects occurs, mainly at the level of the digestive system, where undigested saccharides become a food source for microbial fermentation.

Zavesca^®^ (**4**, Miglustat also known as NBDNJ; Figure 3) is the *N*-butyl DNJ derivative licensed for the treatment of severe lysosomal storage disorders (LSD), i.e., Gaucher [32,33] and Niemann–Pick type C diseases [34]. It reversibly inhibits glucosylceramide synthase, the enzyme catalyzing the first committed step of glycosphingolipid synthesis, lowering the build-up of glucosylceramide [35]. It is not surprising that Zavesca, being structurally similar to Glyset, exhibited the same undesired effects; nevertheless, their successful presence in the pharma market inspired and stimulated further research towards the identification of the second generation leads from iminosugars (Seglins), represented by novel iminosugars endowed with enhanced potency and higher specificity for the target enzymes.

Adverse effects associated with the use of iminosugars have been partially reduced by using them as active site-specific chaperons (ASSC), i.e., small inhibitors competitively interacting with unfolded enzymes, used in sub-inhibitory concentrations, that are able to restore the mutated enzyme allowing the correct trafficking from ER to lysosome.

After the first unfruitful attempts encountered with Plicera^®^ (**5**, *iso*fagomine, Afegostat; Figure 3) as a pharmacological chaperone (PC) in the treatment of Gaucher disease [36,37,38], the approval in 2016 of Galafold^®^ (**6**, deoxygalactonojirimycin, Migalastat; Figure 3) for the therapy of Fabry disease (FD) [39,40,41,42] opened the way to the use of iminosugars as PCs in LSD. Indeed, Migalastat was able to restore the proper conformation of mutated alpha-galactosidase A (α-GalA), the enzyme responsible for the accumulation of globotriaosylceramide in the lysosomal compartment in FD patients.

The success of iminosugars as PCs is that even when they act by binding to the catalytic site and as inhibitors of the target enzyme, in lysosomes, they behave as reversible binders thanks to the reduced binding affinity at low pH and high substrate concentration and thus favor substrate turnover. However, the main limit to the therapeutic efficacy of the PC approach remains the need to finely tune the chaperoning activity with enzyme inhibition.

On the other hand, issues related to competitive inhibition can be overcome with iminosugars acting as allosteric or nonactive site binders able to stabilize mutant proteins and promote ER trafficking, allowing at the same time simplified administration procedures and an increase in enzyme activity [43]. This approach has found some successful applications in lysosomal rare disease pharmacological treatments [44,45]. Moreover, over the last decade, iminosugar therapeutic potential has also been evaluated in the management of another rare disease, cystic fibrosis.

## 2. Iminosugars in Cystic Fibrosis

Cystic fibrosis (CF) is an autosomal recessive inherited disease that leads to multi-system organ dysfunction, mainly affecting respiratory, digestive and reproductive systems [46,47,48].

CF mostly involves epithelial cells, causing damage to the lungs, sinuses, pancreas, liver, bile ducts, intestines, reproductive tract, bones and sweat glands. Respiratory disease is considered the most serious consequence of CF. Functional defects of the cystic fibrosis transmembrane conductance regulator (CFTR) protein, a cAMP-activated chloride channel that controls ion and water content in epithelial cells, are caused by mutations in the *CFTR* gene [49].

In the lungs, the defective CFTR causes dehydration of the airway surface liquid and therefore hyperviscose mucus that triggers a cascade of pathological events leading to chronic infections and inflammation of the airways. This then leads to irreversible lung damage and fibrosis, which represent the major causes of mortality in CF patients.

Available CF therapeutic treatments are based on the use of CFTR modulators, mucolytics, antibiotics to counteract bacterial colonization and lung infections and dietary management. On the other hand, high-dose ibuprofen, a non-steroidal anti-inflammatory drug, remains one of the most effective intervention lines to fight the exaggerated inflammatory response that causes chronic inflammation.

Currently, researchers are working on different approaches, some of them aimed to handle the basic molecular defect in CF, by restoring proper function to the CFTR protein or correcting its production process so that a normal protein can be build up [50,51,52,53,54], others directed to controlling the clinical manifestations of the diseases, including inflammation, infection and mucociliary clearance, mostly for patients with irreversible lung damage [55,56,57,58,59]. The iminosugar class has representative examples in both fields of application and the results obtained in the last decades have been examined below.

## 3. Rescuing the Activity of Defective CFTR: Iminosugars as Correctors

*CFTR* mutations have been grouped into six different classes [49] on the basis of the molecular mechanisms leading to the CFTR protein malfunction: Class I mutations cause the formation of incomplete length proteins with total loss of their activity. Class II mutations produce defective CFTR protein processing and trafficking to the plasma membrane. Class III mutations are relatively rare; the CFTR protein is properly synthesized, transported and fused into apical cell membrane, but it is characterized by altered gating properties and reduced open probability of the ion channel. Class IV, V and VI mutations are respectively characterized by defective chloride conductance, diminished CFTR transcription levels and by accelerated turnover at the cell surface.

Even if about 2000 mutations can affect the CFTR protein, F508del (class II) represents the most frequent mutation, carried by about 90% of CF patients. F508del mutation causes CFTR misfolding and its retention in the ER where the “quality control machinery”, termed endoplasmic reticulum-associated degradation (ERAD), provides for its rapid proteasomal degradation. In addition to trafficking defect, F508del-CFTR also presents characteristic defects of classes III and IV with altered gating of the channel and reduced membrane stability of the rescued protein.

Over the last two decades, many efforts have been devoted to the development of therapeutic agents, namely CFTR modulators, addressed to enhance CFTR intracellular trafficking (correctors), CFTR ion channel function (potentiators) and to increase the amount of CFTR protein at the apical cell membrane, or improve the availability of CFTR for the interaction with other CFTR modulators (amplifiers) [50,60,61]. Even though only four CFTR modulator-based therapies are currently in clinical use (Kalydeco^®^ [62], Orkambi^®^ [63], Symdeko^®^/Symkevy^®^ [64] and Trikafta^TM^ [65]), several small molecules have been demonstrated to be able to restore the expression and/or function of the mutated CFTR [46,54,66]. Regarding iminosugars, attention has been focused on the trafficking defect of F508del-CFTR, whose correction may be achieved through direct modulation of the protein folding (pharmacological chaperones) or acting on enzymes involved in the protein proteostasis pathway [46,60,67].

### 3.1. Iminosugars as CFTR Correctors: NBDNJ and beyond

Among bioactive iminosugar-based compounds, Miglustat (NBDNJ, **4**) has been identified as the first representative example showing interesting pharmacological potential for the treatment of CF.

**Scheme 1:**
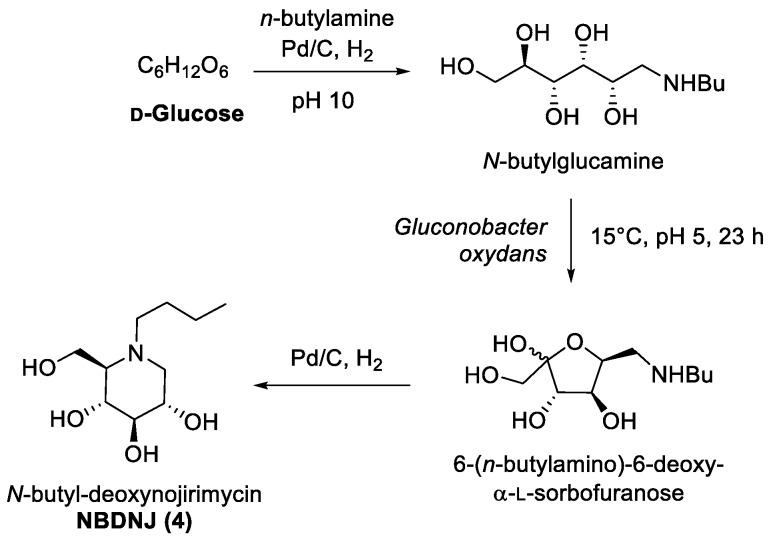
Chemo-enzymatic synthesis of NBDNJ (reproduced with permission from [1]).

Because of its involvement in a variety of therapeutic contexts, a plethora of synthetic routes to NBDNJ and most generally to *N*-alkyl DNJ derivatives have been reported [68,69,70,71]. Large-scale access to NBDNJ is achieved starting from glucose and butylamine through a one-pot chemo-enzymatic route, involving the selective oxidation of C2 by *Gluconobacter oxydans* [72] and the subsequent ring expansion under reductive conditions (Scheme 1) [1,73]. The synthesis was developed by Searle/Monsanto in view of the evaluation of NBDNJ in anti-HIV clinical trials [74].

**Figure 4:**
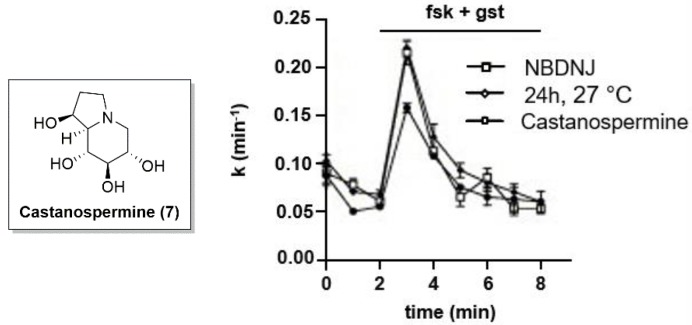
Rescue of functional F508del-CFTR (deletion of F508 residue in the cystic fibrosis transmembrane conductance regulator) activity by NBDNJ, low temperature or **7** as resulting by iodide efflux experiments. This figure is adapted with permission from [75].

Early studies were carried out by Becq et al. and were focused on the capacity by **4** to restore the trafficking of F508del-CFTR protein by inhibiting the trimming of ER glucosidases [75]. Iodide efflux experiments, performed in human airway epithelial cells (CF15) [76], highlighted a significant F508del-CFTR rescue for **4**. The effect was superimposable to that obtained by low-temperature treatment [77] (Figure 4). In the same study, a positive response was also observed for the bicyclic iminosugar castanospermine (**7**), although to a lesser extent than NBDNJ. A similar correction effect was observed for NBDNJ in different delF508-CFTR-expressing human cell lines [75,78]. The iminosugar **4** was also found to restore 12% mature CFTR and 55% of wild type chloride secretion in intestinal cells of F508del mice [75]. Both **4** and **7** were found to prevent delF508-CFTR/calnexin interaction in the ER. Due to the inhibition of the cleavage process of terminal glucose residues in the nascent protein in the ER by means of glucosidase inhibition, it was hypothesized that both iminosugars could interfere with the activity of calnexin, preventing UPP (ubiquitin-proteasome pathway)-mediated degradation of the misfolded CFTR protein [75].

The rescue of defective F508del-CFTR trafficking and function, observed after short-term (2–4 h) treatment of CF cells with **4**, was also accompanied by a normalization of other CFTR-dependent functions affected in CF including Na^+^ transport [79] and Ca^2+^ homeostasis [80]. The same beneficial effects were observed for chronic treatments (up to two months) with low concentration of **4**, providing the first evidence of the reversible rescue of a respiratory CF cell toward a non-CF like phenotype [81].

The ability by **4** to correct ion transport abnormalities was also assessed in F508del mice [82]. Lubamba and co-workers evaluated the effect of in vivo nasal delivery of **4**, measuring the transepithelial potential difference (PD) across the nasal mucosa. These studies demonstrated the improvement of both sodium and CFTR-dependent chloride transport by nasal delivery of picomolar doses of **4**. In addition, NBDNJ was also found to have beneficial effects on bone mass and microarchitecture in F508del-CFTR mice, suggesting a potential application of the drug in the therapeutic treatment of CF-related bone diseases [83].

Despite the strong in vitro and preclinical evidences, phase II clinical trials failed to demonstrate a significant effect by NBDNJ in chloride transport in CF patients [84]. Even though it was suggested that a longer exposure period might be effective [83,85], these results hampered further evaluation of the molecule and no more clinical data have been reported so far.

### 3.2. N-alkyl DNJ Derivatives: Studying the Role of Lipophilicity on the Rescue of F508del-CFTR Activity

**Scheme 2:**
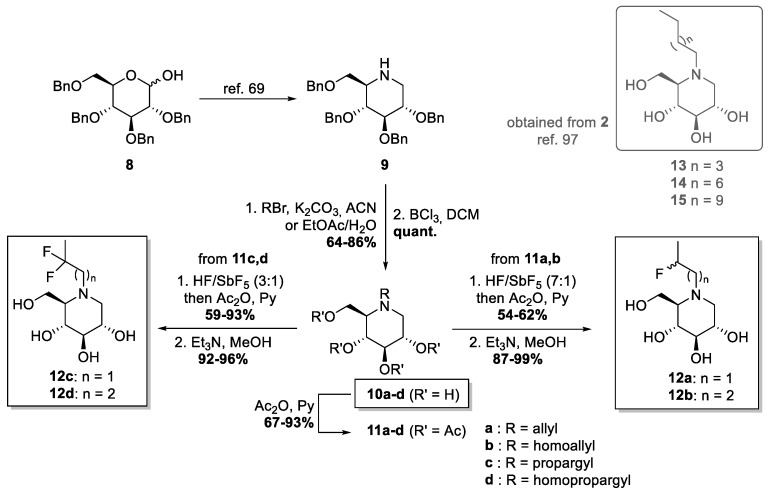
Synthesis of fluorinated and unsaturated *N*-alkyl DNJ derivatives [88].

The results obtained with NBDNJ inspired a variety of structural modifications, which were proposed with the aim to obtain more selective and potent derivatives [1,86,87]. Cendret et al. evaluated a variety of *N*-substituted iminosugars as F508del-CFTR correctors [88]. The synthesis of all compounds involved the use of tetra-*O*-benzyl-deoxynojirimycin (**9**) (Scheme 2). The latter was in turn obtained from commercially available tetra-*O*-benzyl d-glucopyranose (**8**), using the scalable procedure reported by Wennekes et al. [69]. Direct *N*-alkylation of **9** provided unsaturated derivatives **10** [89]. Functionalization of double and triple bonds of **10a–d** was then accomplished through hydrofluorination of the corresponding tetra-acetate derivatives **11a–d**, exploiting HF/SbF_5_ as the fluorinating agents in various combination ratios (Scheme 2) [90,91,92].

**Figure 5:**
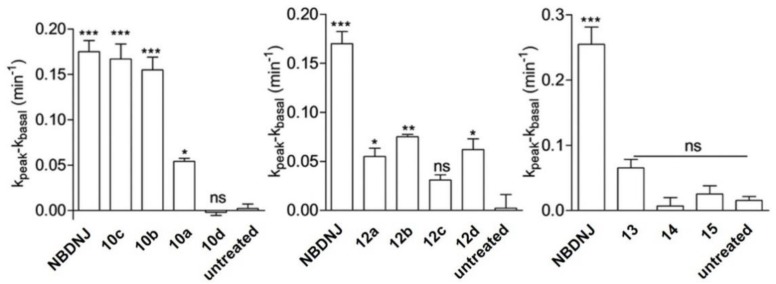
F508del-CFTR correction by saturated, unsaturated and fluorinated *N*-alkyl DNJ derivatives. * *p* < 0.05; ** *p* < 0.01; *** *p* < 0.001; ns, not significant. This figure is republished with permission of the Royal Society of Chemistry, from [88]; permission conveyed through Copyright Clearance Center, Inc.

DNJ derivatives **10** and **12a–d** were examined for the correction capacity of the CFTR function in CF-KM4 cells [93] using iodide efflux experiments (Figure 5) [94]. Iminosugars **13–15** bearing saturated alkyl chains were also considered [8,95,96,97]. No significant rescue capacity of F508del-CFTR activity was observed for most iminosugars, including those bearing longer saturated alkyl chains (**13–15**) than NBDNJ, as well as those with alkyl chains having fluorine atoms (**12a–d**). On the other hand, *N*-homoallyl DNJ (**10b**) and *N*-propargyl DNJ (**10c**) showed a remarkable correction effect, which was comparable to that of NBDNJ (Figure 5).

**Figure 6:**
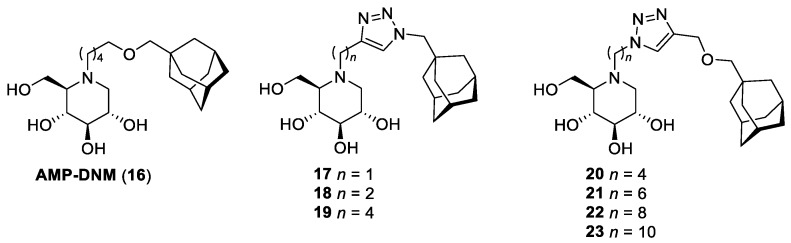
AMP-DNJ and the corresponding triazole-containing derivatives [98].

The above results are broadly in line with those reported by Guisot et al., who conceived to evaluate a library of DNJ derivatives **17–23** (Figure 6), in which triazole-bearing alkyl chains were introduced to link an adamantane moiety to the iminosugar core [98]. This project stands on the intriguing therapeutic potential of AMP-DNM (**16**) [99,100,101,102], even though it must be noted that, to the best of our knowledge, the latter has never been tested as a CFTR corrector.

**Scheme 3:**
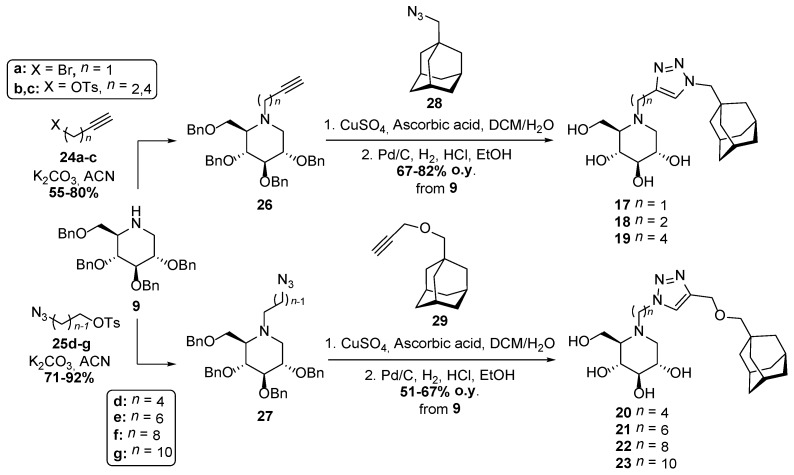
Synthesis of DNJ-adamantane conjugates [98].

Tetra-*O*-benzyl-DNJ (**9**) was derivatized with alkyl chains bearing an alkynyl or an azido terminal moiety, leading to *N*-alkyl iminosugars **26**,**27** (Scheme 3).

The subsequent coupling reaction of the latter with the corresponding click complements bearing an adamantane moiety (azide **28** or alkyne **29** [103]) provided, after the final deprotection, the target compounds **17–23** [104,105].

**Figure 7:**
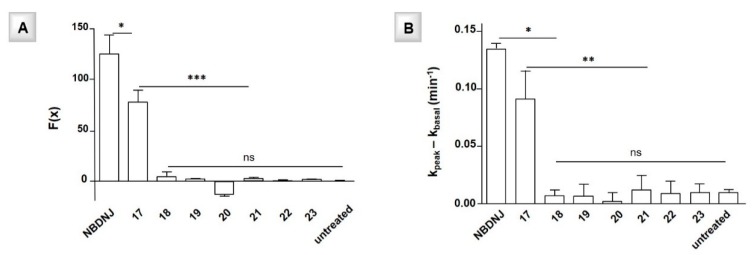
(**A**) Functional evaluation of F508del-CFTR by DiSBAC2(3) assay in CF-KM4 cells treated or not with iminosugar (100 μM, 2 h). (**B**) F508del-CFTR was assayed with the iodide efflux technique in the presence of forskolin/genistein. * *p* < 0.05; ** *p* < 0.01; *** *p* < 0.001; ns, not significant. This figure is republished with permission of the Royal Society of Chemistry, from [98]; permission conveyed through Copyright Clearance Center, Inc.

Among the synthesized DNJ derivatives **17–23**, only the shortest spacer-containing iminosugar **17** showed an interesting effect regarding the rescue of defective F508del-CFTR function in CF-KM4 cells (however, lower than NBDNJ) [93], as highlighted by both single-cell fluorescence imaging (Figure 7A) and iodide effluxes assay (Figure 7B) [76,81]. Interestingly, differently from NBDNJ, compound **17** did not inhibit ER α-glucosidases, suggesting that the mechanism of action dealing with CFTR correction was not related to its inhibitory properties [98].

### 3.3. Iminosugar Click Clusters: Multivalent Effect on the Rescue of CFTR Activity. 

**Figure 8:**
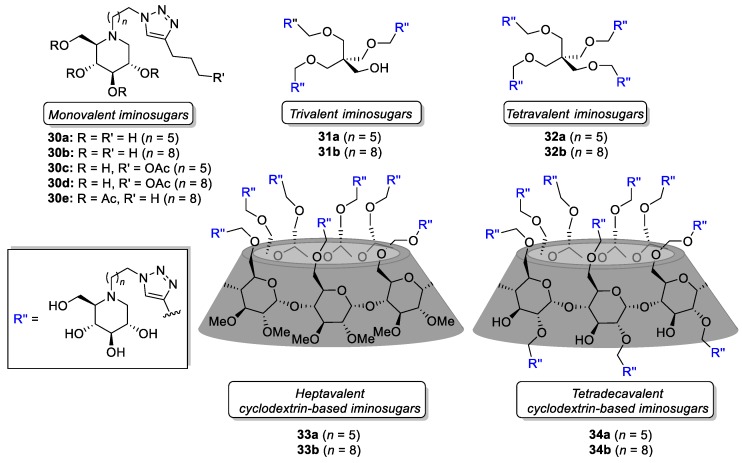
Multivalent iminosugars as F508del-CFTR correctors.

With the aim to improve the pharmacological efficiency of iminosugars as correctors, Compain et al. applied the concept of the multivalent effect on CFTR-defective trafficking [106,107]. Iminosugar clusters already demonstrated their potential on glycosidase inhibition [108,109,110,111], since an increase in the inhibition potency, as well as an improvement in the enzymatic selectivity, was observed as a result of multivalency [108,112,113,114,115]. Accordingly, several iminosugar click clusters with valencies ranging from 3 to 14, previously tested as glycosidase inhibitors [116,117,118] and pharmacological chaperones for the treatment of lysosomal storage diseases [119,120], were evaluated as CFTR correctors (Figure 8) [106].

Two classes of compounds having different valencies and alkyl spacer lengths (C6 or C9) were considered: tri-and tetravalent derivatives **31–32**, built on a penta-erythritol, and hepta- and tetra-decavalent iminosugar-based cyclodextrins **33–34**. In all cases, the molecular systems were conceived to bear *N*-alkyl DNJ derivatives as peripheral ligands, to obtain “super Miglustat” molecules [106].

**Scheme 4:**
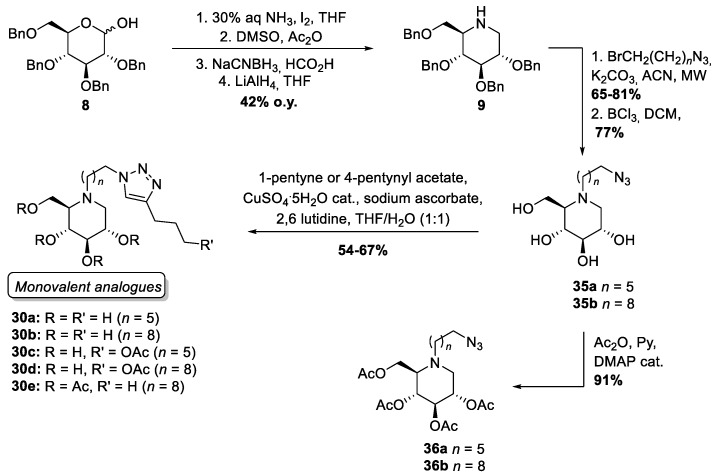
Synthesis of monovalent iminosugar-based analogues [121].

The synthesis involved the formation of amine **9** starting from glucose **8** and the subsequent base-mediated alkylation of **9**, to provide azide-bearing *N*-hexyl and *N*-nonyl DNJ derivatives **35a–b** (Scheme 4) [121]. Click reaction of the last ones with 1-pentyne or 4-pentynyl acetate in presence of CuSO_4·_5H_2_O and sodium ascorbate [104,122] led to monomeric iminosugars **30**, which were used as controls to assess the multivalent effect.

**Scheme 5:**
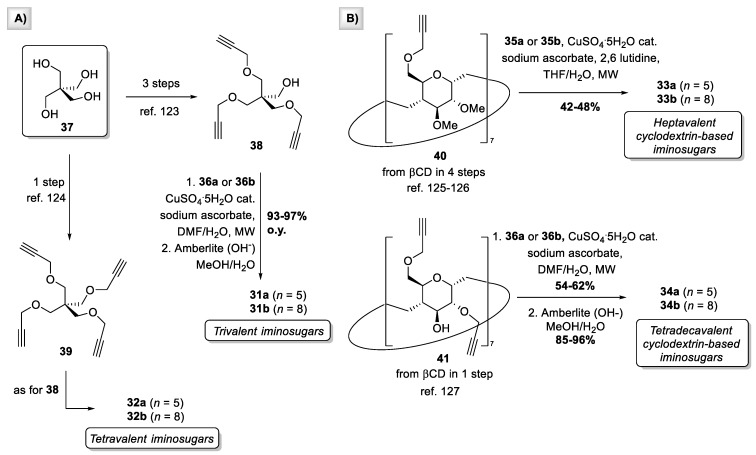
Synthesis of (**A**) tri- and tetravalent iminosugars and (**B**) hepta- and tetra-decavalent DNJ-cyclodextrin iminosugars [106,116].

Iminosugar-based multivalent systems **31–34** were synthesized from both **35** and its peracetylated derivatives **36** (Scheme 5) [106,116]. Cu^I^-catalyzed click coupling of **36** with propargyl ethers **38** and **39,** in turn obtained from tetraol **37** [123,124], gave the tri and tetra-valent iminosugars **31** and **32** (Scheme 5A)**.** Cyclodextrin-based DNJ clusters **33** and **34** were instead prepared through click reaction of **35** and the cyclo-oligosaccharide **40** [125,126] or between **36** and alkyne **41** (Scheme 5B) [127].

**Figure 9:**
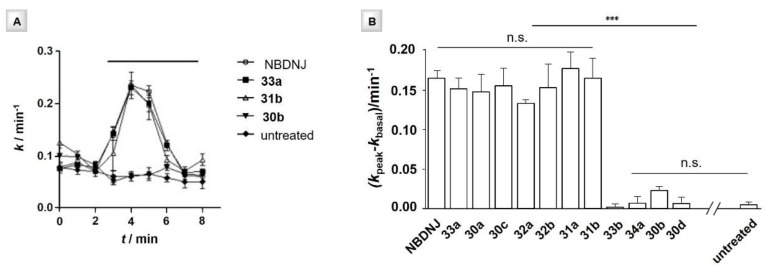
(**A**) Rescue of functional F508del-CFTR activity: iodide efflux curves on untreated or iminosugar-treated CF-KM4 cells in the presence of forskolin/genistein. (**B**) Summary of the functional evaluation of F508del-CFTR: histograms represent the mean (± SEM) of four experiments obtained by the iodide efflux technique in cystic fibrosis (CF) cells untreated or treated (100 μM, 2 h) in the presence of forskolin/genistein. *** *p* < 0.001; n.s., not significant. This figure is adapted with permission from [106]).

Compounds **30–34** were evaluated for their potential to correct F508del-CFTR activity. Iodide efflux on CF cells [94] revealed that monovalent iminosugars **30a** and **30c** and tri-, tetra and heptavalent iminosugars **31a,b**, **32a,b** and **33a** were able to rescue F508del-CFTR function, with an effect comparable to that of NBDNJ (Figure 9).

When evaluated for the correction efficiency, trivalent iminosugars **31** demonstrated the highest activity (Table 1). Particularly, **31a** and **31b** (entries 4 and 8, respectively) largely surpassed the rescuing effect of NBDNJ (entry 1); **31b** was found to be 1000-fold more efficient than the monovalent reference **30b** (entry 7), revealing a significant multivalent effect in the correction of CFTR activity (Table 1). Preliminary mechanistic studies based on a free oligosaccharides (FOS) analysis performed in HL60 cells indicated that, contrarily to the initial assumptions, the strong CFTR corrector activity observed for trivalent iminosugar **31b** was not due to a multivalent effect in the inhibition of ER α-glucosidases I and II. In addition, mono- and multivalent iminosugars **30b** and **31b** both demonstrated a calnexin-dependent mechanism of action. The authors thereby hypothesized that the enhancement in the correction activity could be due to high local concentration effect and/or increased cellular uptake [106].

### 3.4. Pyrrolidine Iminosugars and Pyrrolidine-Containing Bicyclic Iminosugars as Highly Active and Selective CFTR Correctors

**Figure 10:**
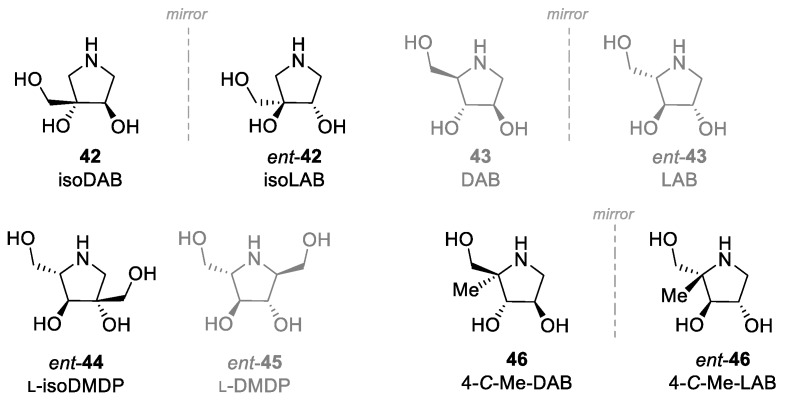
Carbon-branched pyrrolidine iminosugars.

Along with polyhydroxylated piperidines, pyrrolidines are among the most representative members in the iminosugar family. Because of their excellent inhibition potential against a variety of glycosidases [128,129,130], including those residing in the ER [5,131,132], pyrrolidine iminosugars have been considered as modulators in CF. In a series of examples aimed to explore the glycomimetic properties of a series of *arabino*-configured pyrrolidines, Fleet and co-workers evaluated a variety of enantiomerically pure carbon-branched pyrrolidine iminosugars as F508del-CFTR correctors, namely 1,4-dideoxy-2-*C*-hydroxymethyl-1,4-imino-d-threitol (isoDAB, **42**) and its enantiomer 1,4-dideoxy-2-*C*-hydroxymethyl-1,4-imino-l-threitol (isoLAB, *ent*-**42**), 1,4-dideoxy-2-*C*-hyroxymethyl-1,4-imino-l-arabinitol (l-isoDMDP, *ent*-**44**), 1,4-dideoxy-1,4-imino-4-*C*-methyl-d-arabinitol (4-*C*-Me-DAB, **46**) and its enantiomer (4-*C*-Me-LAB, *ent*-**46**) (Figure 10).

**Scheme 6:**
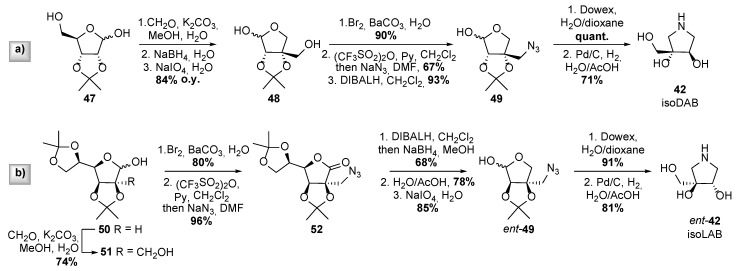
Synthesis of (**A**) isoDAB and (**B**) isoLAB [136].

Research on isoDAB and isoLAB took place in the frame of a SAR study involving the evaluation of inhibitory properties of natural 1,4-dideoxy-1,4-imino-d-arabinitol (DAB, **43**) and its enantiomer 1,4-dideoxy-1,4-imino-l-arabinitol (LAB, *ent*-**43**) against several glycosidases [128,133,134,135]. From a synthetic standpoint, starting from acetonide d-ribose **47**, a Ho crossed aldol reaction allowed the introduction of the branching hydroxymethyl group on the pyrrolidine scaffold. Subsequent reduction and oxidative cleavage led to lactol **48**, which was in turn converted into the azido-lactol **49**. The subsequent reduction of the azido group eventually enabled intramolecular rearrangement, to give the pyrrolidine isoDAB (**42**; Scheme 6A). Similarly, its enantiomer isoLAB (*ent*-**42**) was prepared by a synthetic pathway from diacetonide d-mannose (**50**; Scheme 6B) [136,137].

**Figure 11:**
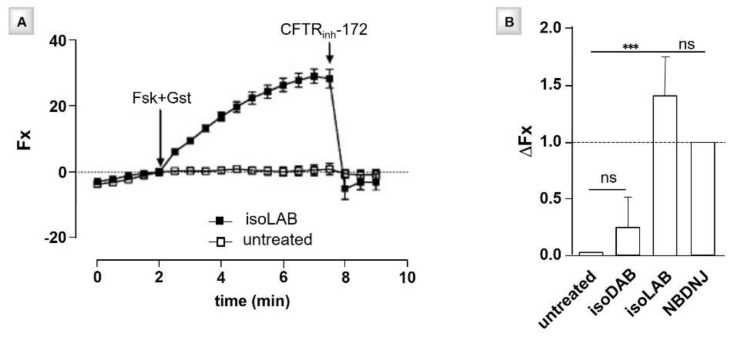
Functional evaluation of F508del-CFTR activity by single-cell fluorescence imaging assay in CF-KM4 cells treated with isoLAB (100 μM, 2 h). (**A**) Time courses obtained with untreated cells or with cells treated with isoLAB in the presence of forskoline/genistein. (**B**) Histograms summarizing the results collected from separate experiments. *** *p* < 0.001; ns, not significant. This figure is reprinted from [136], with permission from Elsevier.

The ability of isoLAB to restore the function of the F508del-CFTR protein in CF-KM4 cells [93] was observed, as indicated by the increase of recorded fluorescence signal after CFTR stimulation in the presence of *ent*-**42** and its sensitivity to the CFTR inhibitor CFTR_inh_-172 (Figure 11A). IsoLAB resulted in a more potent corrector than NBDNJ, while isoDAB displayed a much more limited effect (Figure 11B). Notably, isoLAB showed no significant inhibition of glycosidases, with a significant perspective benefit deriving from limited side effects [136].

**Scheme 7:**
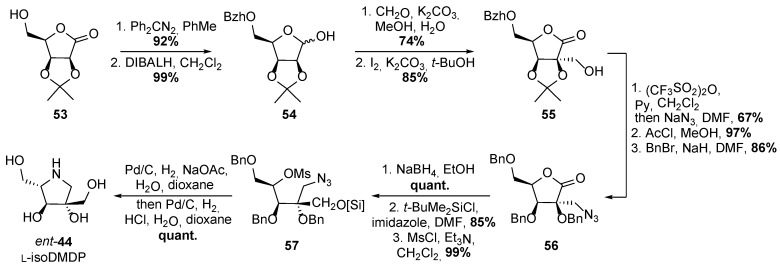
Synthesis of l-isoDMDP [139].

Similarly to isoDAB/isoLAB, research on l-isoDMDP was inspired by the powerful and selective inhibitory ability of l-DMDP against α-glucosidases [138]. The synthesis of l-isoDMDP was based on a sequence of 11 reaction steps starting from d-lyxonolactone acetonide (**53**), in turn obtained from d-galactose. Even in this case, the introduction of the branching hydroxymethyl group was performed by means of the Ho reaction, enabling the conversion of hemiacetal **54** into lactone **55.** After azido group introduction in **56**, pyrrolidine ring closure was accomplished through a procedure involving elaboration of the sugar core and reductive amination, to provide the target l-isoDMDP (Scheme 7) [139].

**Figure 12:**
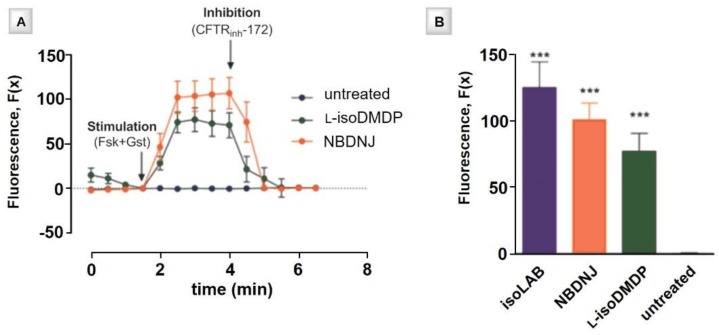
Effect of l-isoDMDP, isoLAB and NBDNJ on the activity of F508del-CFTR in human airway epithelial CF-KM4 cell line, assessed by single-cell fluorescence imaging. (**A**) Cells were incubated with the iminosugars (100 μM, 4 h) in the presence of forskolin/genistein, followed by inhibition with CFTR_inh_-172. (**B**) Histograms summarizing the results collected from separate experiments. *** *p* < 0.001. This figure is adapted with permission from [139]. Copyright (2013) American Chemical Society.

The potential of l-isoDMDP on the rescue of defective F508del-CFTR function was evaluated in CF-KM4 cells and compared with the corrector effect of NBDNJ and isoLAB (Figure 12). l-isoDMDP showed a significant correction effect, albeit lower than either NBDNJ or isoLAB. As for isoLAB, the molecule did not show significant inhibition of ER-resident α-glucosidases I and II.

Differing from previous examples, branched pyrrolidines 4-*C*-Me-DAB and 4-*C*-Me-LAB, in which a methyl group is introduced at C4 position of DAB and LAB (Figure 10), exhibited no correction effect on the functional properties of the defective CFTR [131].

Dehoux et al. investigated the potential of some carbon-branched pyrrolizidine iminosugars [140,141], i.e., the four diastereoisomers of transalpinecine (±**64**) and the two diastereoisomers of the epoxide subulacine (±**62** and ±**63**), as F508del-CFTR correctors.

**Scheme 8:**
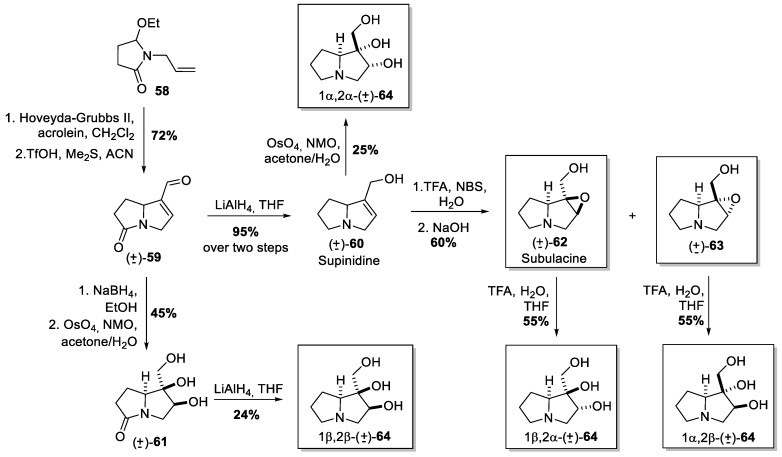
Synthesis of carbon-branched pyrrolizidines [143].

The synthesis of all compounds involved an intramolecular acid-mediated Morita–Baylis–Hillman reaction as the key step (TfOH/Me_2_S) [142], enabling the formation of the bicyclic compound ±**59** from a pyrrolidine scaffold and thereby the access to racemic supinidine (±**60**) after reduction of the aldehyde function (Scheme 8). Then, double bond epoxidation accomplished via bromohydrin formation led to subulacine ±**62** and its diastereoisomer ±**63**, while the four diastereoisomers ±**64** were obtained by epoxide-ring opening of ±**62** and ±**63** under acidic conditions (TFA/H_2_O) or double bond OsO_4_-catalysed *cis*-dihydroxylation of ±**59** or ±**60** [143].

**Figure 13:**
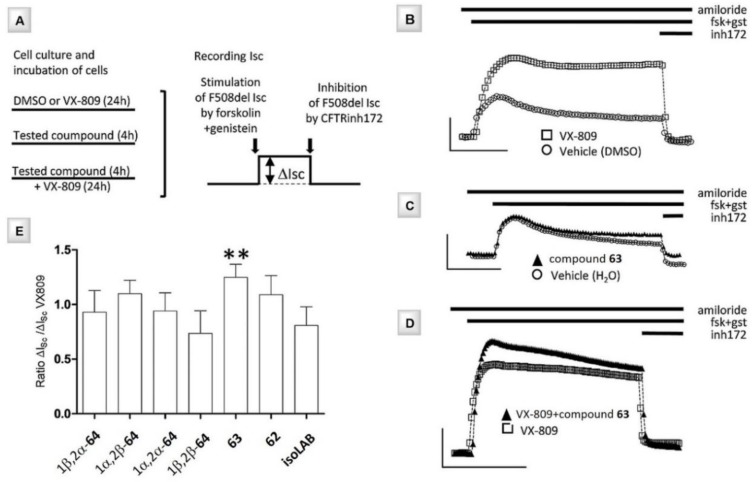
Transepithelial short-circuit currents (Isc) recorded in F508del-CFTR-expressing CFBE cells. (**A**) Protocols used and model trace indicating ΔIsc. (**B**–**D**) Tracings of Isc recorded with CFBE cells incubated with: (**B**) DMSO or VX-809 (10 μM) (24 h); (**C**) water or **63** (100 μM) (4 h); (**D**) VX-809 (10 μM, 24 h) or VX-809+**63** (100 μM, 4 h). F508del-CFTR was activated by application of CTR activators and inhibited by CFTRinh172. (**E**) Summary of the ratio ΔIsc/ΔIsc VX-809 for each compound; ** *p* < 0.01. A ratio above 1 means a potentiation. This figure is adapted with permission from [143].

The rescue of defective protein was evaluated by recording short-circuit currents (Isc) in F508del-CFTR-expressing human airway epithelial cells CFBE (Figure 13). 

Pyrrolizidine iminosugars ±**62**, ±**63** and ±**64** were evaluated either alone or in combination with the corrector VX-809 (Lumacaftor) [144,145], which is one of the two components of the currently marketed drug Orkambi^®^ (Lumacaftor/Ivafactor). Compared to the vehicle, VX-809 stimulated F508del-Isc (Figure 13B) while compound **63** had no effect (Figure 13C). However, when **63** was co-administered with VX-809, an increase in F508del-Isc stimulation was observed (Figure 13D,E), indicating that the compound was able to improve the activity of F508del-CFTR corrector VX-809. Conversely, none of the other pyrrolizidines showed a correction activity either alone or in combination with VX-809 (Figure 13E) [143].

**Scheme 9:**
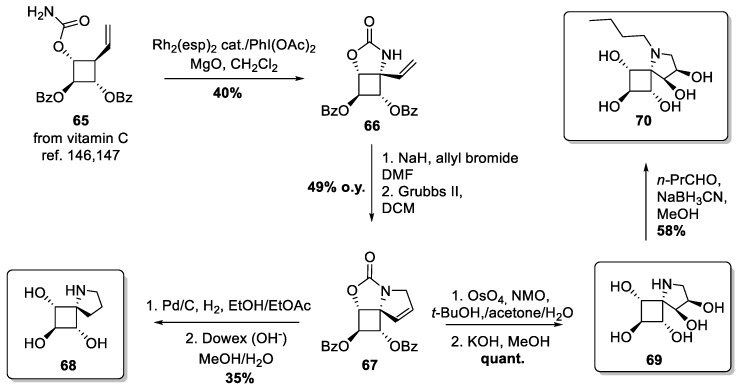
Synthesis of conformationally constrained iminosugars **68–70** [147,150].

Compain et al. developed a novel class of conformationally constrained iminosugars **68–70**, based on four-membered ring-containing spirocycles (Scheme 9) [146,147]. In this case, the rigid bicyclic system was conceived with the idea to reproduce the structure of bioactive iminosugars (e.g., DNJ and castanospermine), while more closely resembling the conformation of the substrates of carbohydrate-processing enzymes in their transition states [148,149]. The synthesis of **68–70** relied on the Rh(II)-catalyzed C(sp^3^)-H amination of carbamate **65** (in turn obtained from vitamin C [150]) enabling a stereoselective C-N bond formation in **66** [151,152]. Subsequent *N*-allyl carbamate formation followed by ring-closing metathesis using Grubbs II catalyst provided intermediate **67**. Hence, double-bond hydrogenation of alkene **67** followed by basic hydrolysis of both benzoyl groups and carbamate ring provided **68**, while *cis*-dihydroxylation of **67** under Upjohn conditions led to diol **69**. *N*-alkylation of the latter under standard reductive amination conditions (butanal/NaBH_3_CN) gave access to **70**, which was conceived to act as a constrained NBDNJ analogue [147].

**Figure 14:**
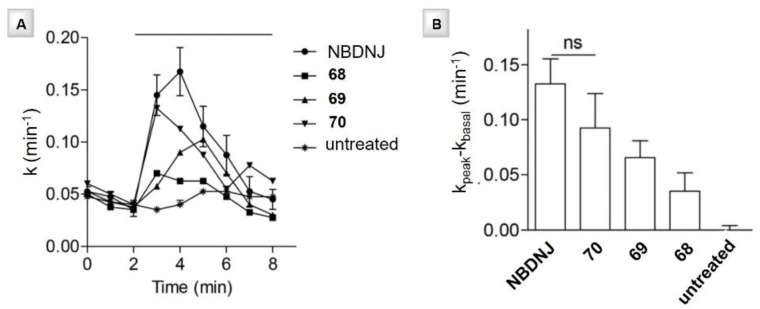
(**A**) Rescue of functional F508del-CFTR activity. Iodide efflux curves on untreated or iminosugar-treated F508del-CFTR stably transfected HeLa cells in the presence of forskolin/genistein. (**B**) Summary of the functional evaluation of F508del-CFTR. Histograms represent the mean (± SEM) of four experiments obtained by the iodide efflux technique in CF cells untreated or treated with the iminosugar (100 μM, 4 h). ns, not significant. This figure is adapted from [146], published by the Royal Society of Chemistry.

When evaluated for their ability to rescue the defective F508del-CFTR function in cells [94], iminosugars **68–70** showed a positive effect (Figure 14), albeit lower than NBDNJ. Particularly, compound **70** showed the highest activity, suggesting a role of the butyl moiety and the hydroxyl groups for the rescuing activity.

### 3.5. N- and C-Alkyl Azepane Iminosugars as CFTR Correctors 

Although less popular than piperidines and pyrrolidines, azepane iminosugars exhibit interesting properties as either inhibitors [153] or chaperones [154,155] of carbohydrate-processing enzymes. Accordingly, Désiré and co-workers widened the therapeutic potential of azepanes exploring the capacity of a panel of *N*- and *C*- alkyl seven membered iminosugars **71–78** (with alkyl chains ranging from 4 to 12 carbon atoms) to rescue the activity of the defective F508del-CFTR [156].

**Figure 15:**
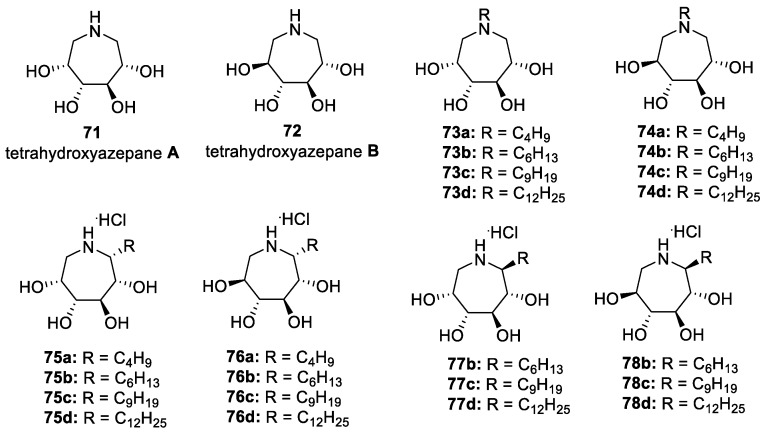
Tetrahydroxylated azepanes evaluated by Désiré and co-workers [156].

**Scheme 10:**
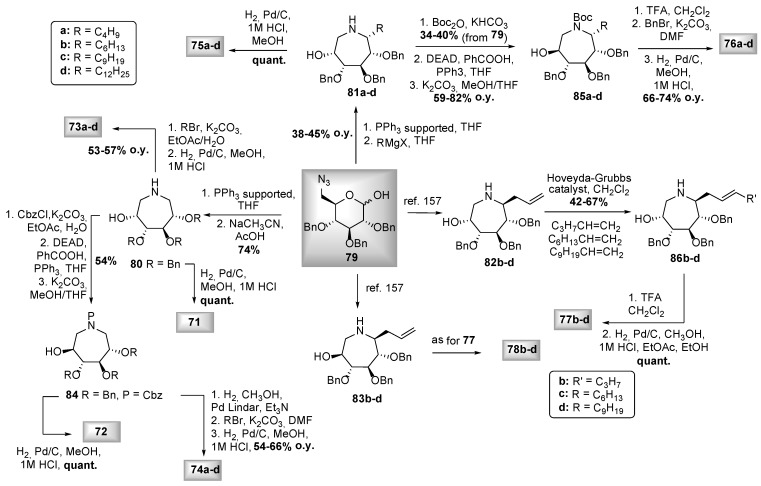
Synthesis of *N*-alkyl and *C*-alkyl azepane iminosugars [156].

Enantiopure iminosugars **71–78** (Figure 15) were synthesized from the protected 6-azido-glucose **79,** exploiting a Staudinger/aza Wittig ring expansion, to build up the seven-membered skeleton providing, depending on reaction conditions, azepane **80** or *C*-azepanes **81–83** [157,158]. Hence, elaboration of **80** led to **71** and **72** and the corresponding *N*-alkylated derivatives **73** and **74,** while azepanes **75** and **76** were obtained from **81**. Synthesis of *C*-azepanes **77** and **78** was accomplished by cross metathesis of *C*-allyl azepanes **82** and **83** in the presence of suitable alkenes (Scheme 10) [156].

**Figure 16:**
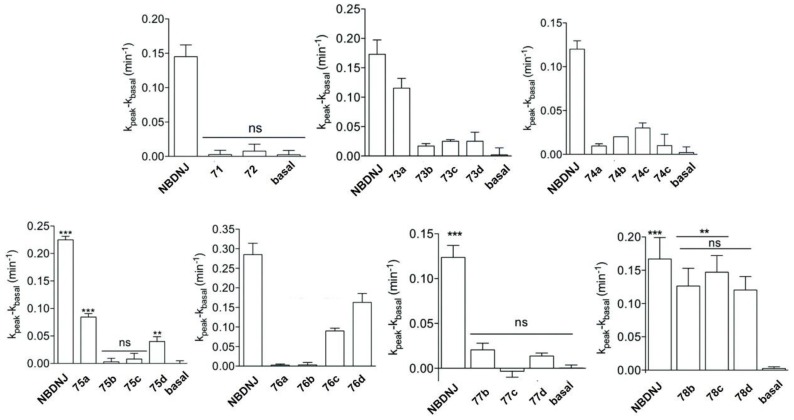
Azepane iminosugars as F508del-CFTR correctors. ** *p* < 0.01; *** *p* < 0.001; ns, not significant. This figure is republished with permission of the Royal Society of Chemistry, from [156]; permission conveyed through Copyright Clearance Center, Inc.

When evaluated as CFTR correctors in CF-KM4 cells, azepanes **71** and **72** and the corresponding *N*-alkylated derivatives **73** and **74** showed no significant activity (Figure 16). On the contrary, *C*-alkylated azepanes **78** were able to rescue the activity of F508del-CFTR, with a correction efficacy comparable to that of NBDNJ, regardless of alkyl chain length. Notably, iminosugars **78** did not inhibit ER α-glucosidases I and II, as revealed by FOS analysis.

The mechanism behind the observed activity was not clear; the authors hypothesized that a binding process to the glycosylated site of the protein could be involved [156].

### 3.6. Combination Studies of CFTR Correctors

**Figure 17:**
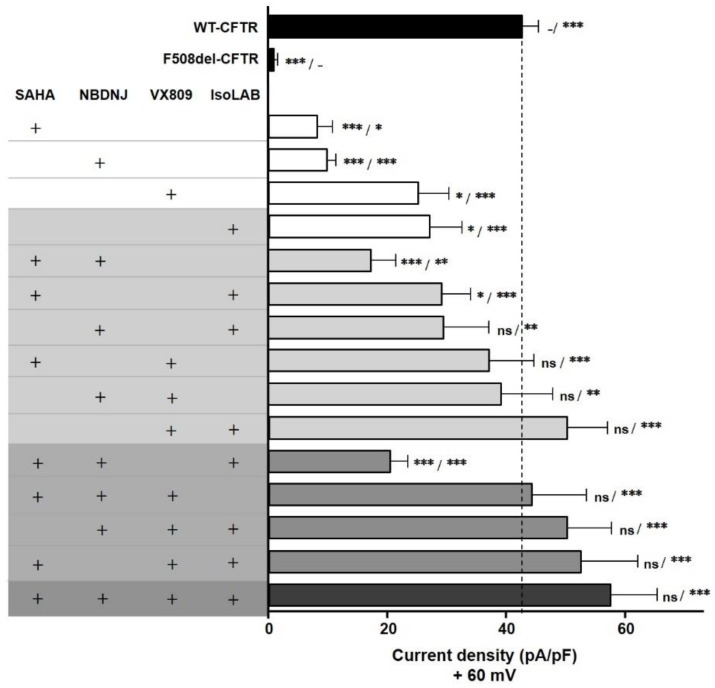
Effect on the F508del-CFTR rescue after application of several correctors individually or in combinations. * *p* < 0.05; ** *p* < 0.01; *** *p* < 0.001; ns, not significant. This figure is adapted with permission from [159].

The capability of both NBDNJ (**4**) and isoLAB (*ent*-**42**) to restore F508del-CFTR function was also assessed evaluating their effect in combination with other correctors [159], based on evidence that an increase of the correction efficiency can be achieved when in combination with small molecules, because of additive or synergistic effects [51,54,160,161,162]. As shown in Figure 17, the use of isoLAB in combination with the corrector VX-809 led to a significant increase of CFTR function. An even higher effect was observed using a combination of the four correctors SAHA (hydroxamic acid [163]), NBDNJ, isoLAB and VX-809.

Most notably, the combination of isoLAB with VX-809 led to the highest level of functional correction and increase of mature c-band of F508del-CFTR proteins.

## 4. Iminosugars as Therapeutic Agents for the Treatment of CF Lung Inflammation 

Despite the advances in CFTR restoration, these therapies are not available for all CF patients and are not able to reverse lung damage in patients with established disease [46,55,57]. Indeed, irreversible lung damage, caused by chronic airway inflammation, is currently the primary cause of morbidity and mortality in CF patients [164,165]. Accordingly, the development of novel and safe anti-inflammatory agents able to preserve lung function still represents a central issue in CF drug discovery [166,167]. With the aim to control excessive inflammation without affecting the host defense against airway CF pathogens, novel targets are currently being considered [58]. In this context, several studies highlight that modulation of sphingolipid (SL) metabolism can be used to control the inflammatory response in CF [168,169], offering novel opportunities for therapeutic applications of iminosugars in CF.

### 4.1. Effect of Iminosugars on the Inflammatory Response to P. aeruginosa: from NBDNJ to Its N-Alkyl Derivatives

**Figure 18:**
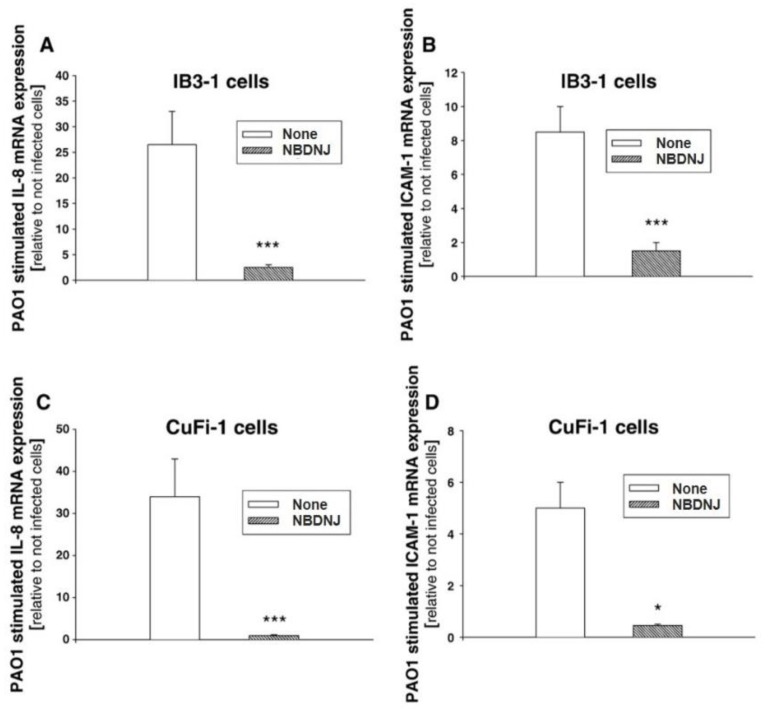
Effect of NBDNJ on the *P. aeruginosa* stimulated IL-8 and ICAM-1 mRNA expression in (**A**,**B**) IB3-1 cells and (**C**,**D**) CuFi-1 cells. Cells were incubated for 24 h in the presence of NBDNJ (200 μM) and infected with PAO1 (50 CFU/cell) for 4 h. * *p* < 0.05; *** *p* < 0.001. This figure was reprinted from [78], with permission from Elsevier.

Early studies on the anti-inflammatory effect of iminosugars in CF were driven by the need to control the inflammatory response to *P. aeruginosa* by modulation of defective CFTR [57,170]. In this context, based on the CFTR rescue ability exerted by NBDNJ [75,79], Dechecchi et al. investigated its anti-inflammatory effect in CF bronchial epithelial cells, measuring the *P. aeruginosa* stimulated inflammatory response [78]. As a result, NBDNJ strongly reduced both IL-8 and ICAM-1 expression in CF cell models IB3-1 and CuFi-1 cells (Figure 18).

**Figure 19:**
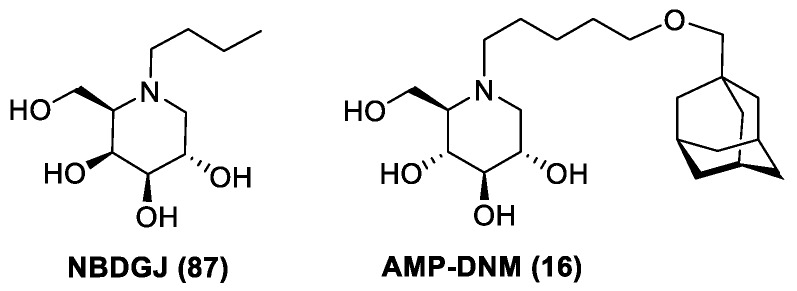
Iminosugars with anti-inflammatory potential.

However, the anti-inflammatory effect was also observed in CF bronchial epithelial cells treated with NBDGJ (**87,** Figure 19), which, differing from NBDNJ, did not restore CFTR function [75,78]. These data, combined with the observed inhibition of IL-8 expression when non-CF bronchial NuLi-1 cells were treated with both NBDNJ and NBDGJ, led the authors to hypothesize that the anti-inflammatory activity exerted by NBDNJ could involve a mechanism other than F508del-CFTR rescue.

**Scheme 11:**
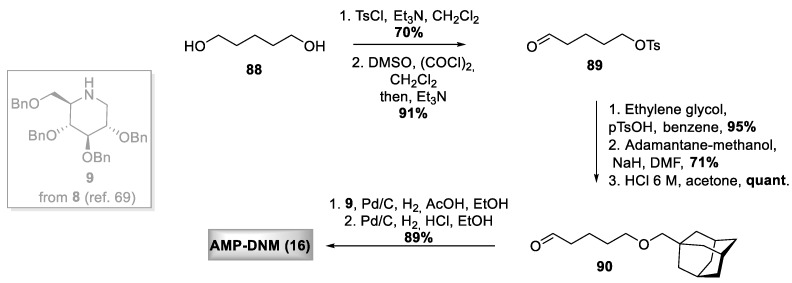
Synthetic route to AMP-DNM [69,70].

**Figure 20:**
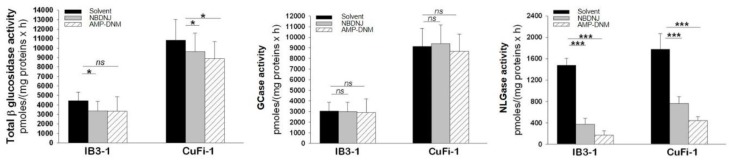
Effect of NBDNJ and AMP-DNM on NLGase inhibition in IB3-1 and CuFi-1 cells infected by *P. aeruginosa.* * *p* < 0.05; *** *p* < 0.001; ns, not significant. This figure is adapted from [173] published by PLOS.

In line with evidence about the involvement of sphingolipid (SL) metabolism in CF pulmonary inflammation and infection [168,169,171], subsequent studies revealed that NBDNJ exerted a significant anti-inflammatory effect both in human bronchial epithelial cells in vitro and in murine models of lung inflammation in vivo by reducing the *P. aeruginosa* induced production of ceramide [56,172]. In-depth studies allowed the association of the anti-inflammatory effect exerted by NBDNJ with the inhibition of the non-lysosomal β-glucosidase 2 (GBA2-encoded NLGase), the enzyme involved in the metabolism of SLs by ceramide production at the plasma membrane level [168,173,174]. Indeed, Loberto and co-workers demonstrated that not only NBDNJ, but also the potent NLGase inhibitor, AMP-DNM (**16**) [99], was able to reduce the *P. aeruginosa* stimulated IL-8 mRNA expression in CF bronchial cells, and that both iminosugars inhibit the NLGase activity in IB3-1 and CuFi-1 cells infected by *P. aeruginosa* without affecting lysosomal β-glucocerebrosidase (GBA1-encoded GCase) activity (Figure 20) [173]. The large-scale synthesis of AMP-DNM was performed using the approach developed by Overkleeft and coworkers, involving the reductive amination of protected DNJ (**9**) with aldehyde **90**, in turn prepared in five reaction steps from 1,5-pentanediol (Scheme 11) [69,70].

These findings support the potential of the enzymes involved in SL metabolism as novel targets for CF lung disease treatment, opening new perspectives for the therapeutic use of iminosugars in CF.

With the idea to combine the anti-inflammatory effect of NBDNJ and AMP-DNM with the increase in potency and selectivity regarding NLGase inhibition exerted by lipophilic iminosugars [96,102,175], Munari and co-workers evaluated the therapeutic potential for CF lung inflammation treatment of the aforementioned AMP-DNM analogues **17–23**. As shown in Table 2, all compounds were found to reduce the expression of IL-8 induced by *P. aeruginosa* infection in CF bronchial cells with IC_50_ values in the nanomolar range (comparable to that of AMP-DNM) [176].

**Figure 21:**
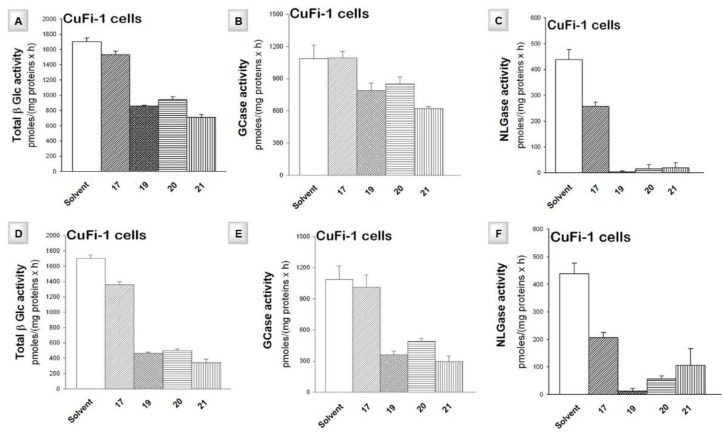
Effect of AMP-DNM derivatives on β-glucosidase activity in CF bronchial cells. CuFi-1 cells were treated with (**A**–**C**) 0.5 μM or (**D**–**F**) 5.0 μM of compound for 1 h before the measurement (adapted from [176]).

The same authors also observed NLGase inhibition in CF bronchial cells treated with AMP-DNM analogues **17, 19–21** (Figure 21), providing further evidence of the anti-inflammatory effect by modulators of GSL metabolism in CF lung disease. Particularly, the iminosugar **17** bearing the shortest alkyl chain as spacer was the weakest NLGase inhibitor, even though it did not affect GCase activity as well as ER α-glucosidases and it was a poor inhibitor of intestinal glycosidases [98]. Due to its selective inhibition towards NLGase combined with its effect as CFTR corrector, **17** represented an interesting candidate to be further explored as a therapeutic agent for CF treatment.

### 4.2. Exploring the Role of the Chirality in the Anti-Inflamatory Effect of N-alkyl DNJ Derivatives

**Figure 22:**
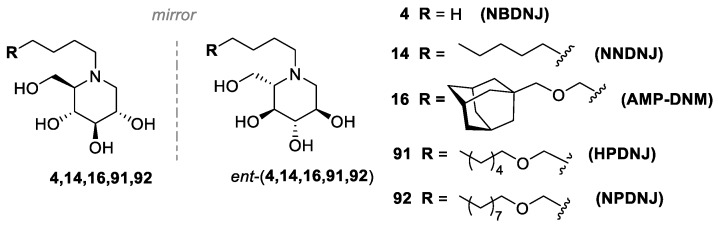
*N*-Alkylated d- and l-DNJ derivatives. This figure is reprinted from [179], with permission from Elsevier.

The chirality of iminosugars demonstrated an important role in their pharmacological properties against carbohydrate-processing enzymes, including NLGase, especially in terms of enzymatic selectivity [45,96,177,178]. In this context, De Fenza et al. explored the inhibition potential of l-*gluco*-configured *N*-alkyl-deoxyiminosugars, *ent*-(**4,14,16,91,92**) (Figure 22) against NLGase, thereby studying the effect of chirality on the anti-inflammatory treatment of CF [179].

**Scheme 12:**
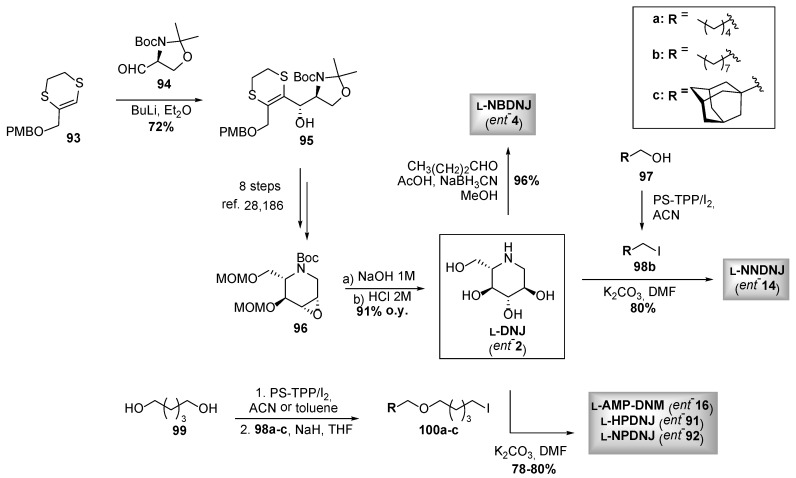
Synthesis of *N*-alkylated l-DNJ derivatives *ent*-(**4,14,16,91,92**) [179,186].

**Figure 23:**
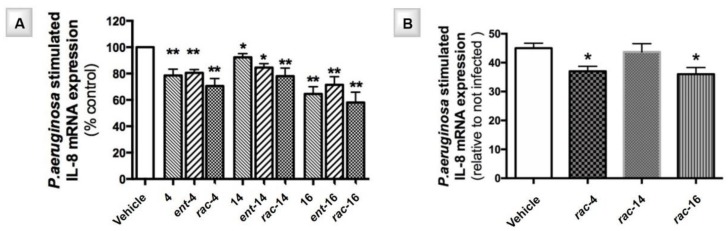
(**A**) Effect of sugar chirality of iminosugars **4,14,16** and *ent*-(**4,14,16**) on the inflammatory response to *P. aeruginosa* in CF bronchial epithelial cells. CuFi-1 cells were treated with the compounds (0.1 μM) for 1 h before *P. aeruginosa* infection. (**B**) Effect of racemic mixtures on the inflammatory response to *P. aeruginosa* in CF primary cells. CF bronchial primary cells were treated with the racemic mixtures (0.01 μM) for 1 h before *P. aeruginosa* infection. * *p* < 0.05; ** *p* < 0.01. This figure was reprinted from [179], with permission of Elsevier.

The synthesis of l-DNJ derivatives was achieved exploiting a de novo methodology [180,181,182] that relied on the use of a homologating agent **93** [183,184,185]. Coupling reaction of this agent with the l-enantiomer of Garner aldehyde (**94**) led to alcohol **95**, whose further elaboration allowed the obtainment of oxirane **96**. Base-mediated epoxide ring opening of **96** followed by acidic hydrolysis of protecting groups gave stereoselectively access to the l-*gluco* configured iminosugar *ent*-**2** [28,186]. l-NBDNJ (*ent*-**4**), the non-superimposable mirror image of NBDNJ, was obtained by standard *N*-alkylation reaction conditions of *ent-***2**, while preparation of *ent*-(**14,16,91,92**) was performed by *N*-alkylation of l-DNJ with reactive alkyl and alkoxyalkyl iodides (**98b** and **100**), in turn prepared by polymer supported triphenylphosphine (PSS-TPP)-mediated iodination reactions (Scheme 12) [187,188].

l-iminosugars *ent*-(**4,14,16,91,92**) were found to act as NLGase inhibitors, although less efficient than their d-enantiomers, and were found to significantly reduce the inflammatory response induced by *P. aeruginosa* in CuFi cells (Figure 23), either alone or in synergistic combination with their d-enantiomers [179]. Notably, *ent*-(**4**) was not able to act as inhibitor for most glycosidases, showing enzymatic selectivity compared to its d-enantiomer [28].

**Figure 24:**
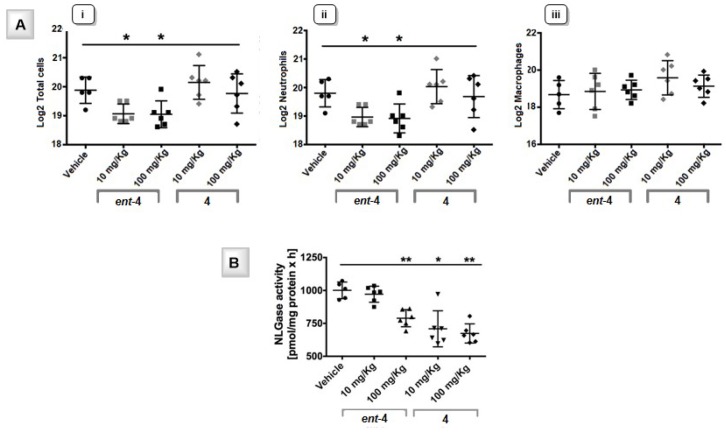
(**A**) Effect of **4** and *ent*-**4** on cells recruited in bronchoalveolar lavage (BAL) of C57Bl/6NCr mice infected by *P. aeruginosa* (i, total cells; ii. neutrophils; iii. alveolar macrophages). (**B**) NLGase activity in the lungs of mice infected by *P. aeruginosa.* * *p* < 0.05; ** *p* < 0.01. This figure was reprinted from [179], with permission from Elsevier.

The anti-inflammatory effect of l-NBDNJ (*ent*-**4**), as well as its inhibition potential against NLGase, were also assessed in C57Bl/6NCr mice infected by *P. aeruginosa*. A reduction of recruitment of neutrophils at a much lower dosage (40-fold) than that of d-NBDNJ [56] (Figure 24A) and a strong inhibition of NLGase activity (Figure 24B) were observed. These data, along with the high enzymatic selectivity associated with the use of l-iminosugars, highlight strong evidence of the therapeutic potential of *N*-alkyl l-iminosugars as anti-inflammatory agents in CF [179].

## 5. Concluding Remarks

Iminosugars represent the most important class of glycomimetics, showing high pharmacological potential in several therapeutic fields, as a result of their excellent ability to interact with carbohydrate-processing enzymes. Over the last years, these properties have been applied to develop diverse therapeutic approaches for the treatment of CF, exploiting the involvement of specific glycosidases in biological processes which are relevant in the pathogenesis of CF. The first and most popular application has been the use of iminosugars as correctors of defective CFTR mutants, with a special focus on the activity of the most common F508del-CFTR. In this case, the iminosugar drug NBDNJ has been identified as the first candidate showing ability to rescue F508del-CFTR function in vitro and in vivo, while the correction effect was not confirmed in clinical trials. Starting from this example, a variety of modifications were explored, highlighting the role of lipophilicity (e.g., unsaturated DNJ derivatives **10;** triazole-bearing AMP-DNM derivatives **17–23**), ring size (e.g., pyrrolidines isoLAB (*ent*-**42**), l-isoDMDP (*ent*-**44**); carbon-branched pyrrolizidine **63**; constrained NBDNJ analogues **68–70;** azepanes **78**) and sugar chirality (e.g., compare isoDAB and isoLAB) on the correction activity. On one hand, enhanced properties as correctors, compared to NBDNJ, were observed in some cases, with isoLAB displaying the most promising pharmacological potential. On the other hand, a variety of these iminosugars did not display glycosidase inhibition, suggesting that the mechanism underlying F508del-CFTR function rescue could be different compared to that initially proposed. Combination studies have also been performed between NBDNJ, isoLAB and other correctors from non-carbohydrate sources, including VX-809, which deserve attention because of the occurrence of additive or synergistic effects.

A second therapeutic approach, which can be perspectively applied for the symptomatic treatment of CF, is based on the anti-inflammatory effect exerted by iminosugars on pathogens (especially *P. aeruginosa*) responsible for lung infection. Starting again from the example of NBDNJ, various structural changes have been investigated, revealing a marked effect depending on compound lipophilicity (AMP-DNM and compounds **17, 19–21**) and sugar chirality [compounds *ent*-(**4,14,16**) and the corresponding racemic mixtures *rac*-(**4,14,16**)] on the anti-inflammatory properties of the corresponding iminosugars, as a consequence of the enhanced and selective NLGase inhibition. Particularly, the analysis of the anti-inflammatory effect of the l-enantiomer of NBDNJ (l-NBDNJ) was also studied in mice models infected by *P. aeruginosa* and a reduction in the recruitment of neutrophils at a much lower dosage (40-fold) than that of its d-counterpart was observed.

Overall, the results reported throughout this review demonstrate that iminosugars have the potential to act as main players for the therapeutic treatment of CF, whether used to restore the functions of the defective protein or to contrast the onset of pro-inflammatory events correlated with the disease. Despite the amount of data so far provided, there is large room for improvement in the field. From a synthetic standpoint, only a limited number of procedures have been applied on an appreciably large scale, and therefore further efforts must be devoted to provide synthetic approaches that are more attractive for industrial applications. Looking at biological assays, the number of candidates identified so far is still relatively small, although current data can provide indications to define future directions for lead identification. This could involve the combination of iminosugars, based on the observation of the synergistic effects so far observed.

## Figures and Tables

**Table 1:** Correction efficiency for multivalent iminosugars (compared to the monovalent congeners) in CF-KM4 cells ^a^ (adapted with permission from [106]).

Entry	Iminosugar	Valency	Spacer Length	EC_50_ (μM)	EC_50_ (mg·L^−1^)
1	NBDNJ	1	-	112	24.6
2	**30a**	1	C_6_	12	4.3
3	**30c**	1	C_6_	20	8.3
4	**31a**	3	C_6_	1	1.1
5	**32a**	4	C_6_	110	158.6
6	**33a**	7	C_6_	71	256.7
7	**30b**	1	C_9_	458	182.5
8	**31b**	3	C_9_	0.5	0.6
9	**32b**	4	C_9_	49	78.9
10	**33b**	7	C_9_	-^b^	-^b^

^a^ CF-KM4 cells were treated for 2 h with 100 mM of compounds in the presence of CFTR activators. ^b^ inactive.

**Table 2:** Inhibition of *P. aeruginosa* stimulated IL-8 mRNA expression in CuFi cells. (Reproduced from [176]).

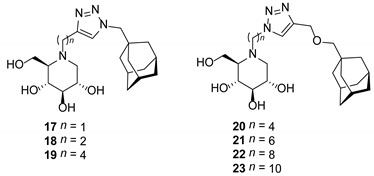			
Entry	Iminosugar	IC_50_ (nM)	M.I. (%)
1	NBDNJ (**4**)	1900	51.5
2	AMP-DNM (**16**)	2	46
3	**17**	12	43
4	**18**	5	23
5	**19**	45	36
6	**20**	35	40
7	**21**	620	36
8	**22**	7	36
9	**23**	2	28

IC_50_: inhibitor concentration that produces 50% inhibition; M.I.: maximal inhibition.

## Abbreviations

ACN	Acetonitrile
AcOH	Acetic Acid
AMP-DNJ	Adamantanemethoxypentyl-DNJ
BAL	Bronchoalveolar lavage
Bn	Benzyl
Bu	Butyl
BVDV	Bovine viral diarrhea virus
Bz	Benzoyl
CF	Cystic Fibrosis
CFTR	Cystic Fibrosis Transmembrane Conductance Regulator
CuAAC	Cu^I^-catalyzed Azide-Alkyne Cycloaddition
βCD	β cyclodextrin
DAB	1,4-dideoxy-1,4-imino-d-arabinitol
DCM	Dichloromethane
DMF	*N*,*N*-dimethylformamide
DMSO	Dimethyl sulfoxide
DNJ	Deoxynojirimycin
EC_50_	Half maximal effective concentration
ENaC	Epithelial Na^+^ channel
ER	Endoplasmatic Reticulum
Et	Ethyl
EtOAc	Ethyl Acetate
EtOH	Ethanol
FOS	Free Oligosaccharides
fsk	Forskoline
GCase	Lysosomal β-glucocerebrosidase
gst	Genistein
HCV	HepatitisC virus
HIV	Human immunodeficiency virus
HPDNJ	Hexyloxypentyl-DNJ
HSV	Herpes Simplex virus
IC_50_	Half maximal inhibitory concentration
ICAM-1	Intracellular adhesion molecule 1
IL-8	Interleukin-8
Isc	Transepithelial short-circuit currents
LAB	1,4-dideoxy-1,4-imino-l-arabinitol
Me	Methyl
MeOH	Methanol
NBDGJ	*N*-butyl-deoxygalactonojirimycin
NBDNJ	*N*-butyl-deoxynojirimycin
NBS	*N*-bromosuccinimide
NJ	Nojirimycin
NLGase	non-lysosomal β-glucosidase 2
NMO	*N*-methylmorpholine *N*-oxide
NNDNJ	*N*-nonyl-DNJ
NPDNJ	*N*-nonyloxypentyl-DNJ
PD	Potential difference
PS-TPP	Polymer Supported Triphenylphosphine
Py	Pyridine
SLs	Sphingolipid
THF	Tetrahydrofuran
TFA	Trifluoroacetic acid
TPP	Triphenylphosphine
UPP	Ubiquitin-Proteasome Pathway
